# Supplementary figures and images for: Domain fusion TLR2-4 enhances the autophagy-dependent clearance of Staphylococcus aureus in the genetic engineering goat
Source: eLife. 2022 Jun 28;11:e78044. doi: 10.7554/eLife.78044 (PMC9239677; doi:10.7554/eLife.78044)

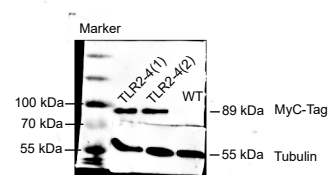

Supplement: Figure 1—source data 1. [file elife-78044-fig1-data1.zip › Figure 1-source data 1.pdf]

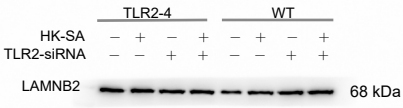

Supplement: Figure 1—source data 2. [file elife-78044-fig1-data2.zip › Figure 1-source data 2 (LAMNB2).pdf]

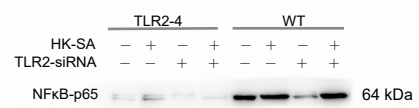

Supplement: Figure 1—source data 2. [file elife-78044-fig1-data2.zip › Figure 1-source data 2 (NFkB-p65).pdf]

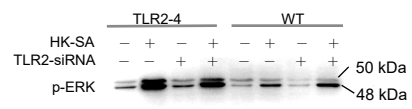

Supplement: Figure 1—source data 2. [file elife-78044-fig1-data2.zip › Figure 1-source data 2 (p-ERK).pdf]

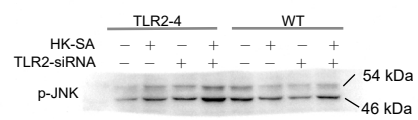

Supplement: Figure 1—source data 2. [file elife-78044-fig1-data2.zip › Figure 1-source data 2 (p-JNK).pdf]

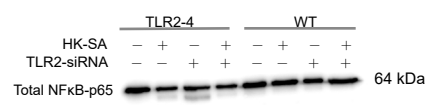

Supplement: Figure 1—source data 2. [file elife-78044-fig1-data2.zip › Figure 1-source data 2 (Total NFkB-p65).pdf]

## Slide 1
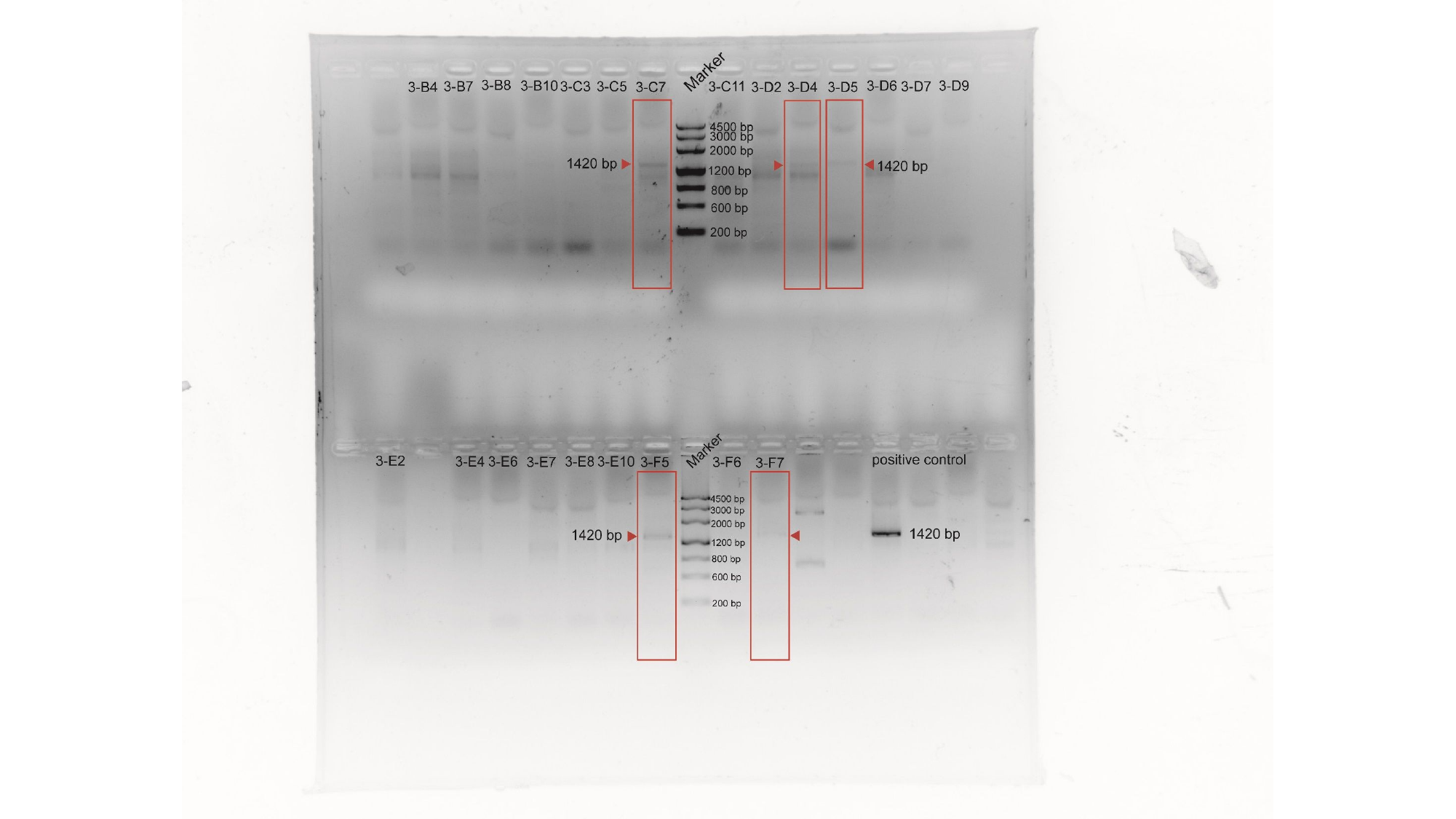

Supplement: Figure 1—figure supplement 1—source data 2. [file elife-78044-fig1-figsupp1-data2.zip › Figure 1-figure supplement 1-source data 2.pptx]

## Slide 1
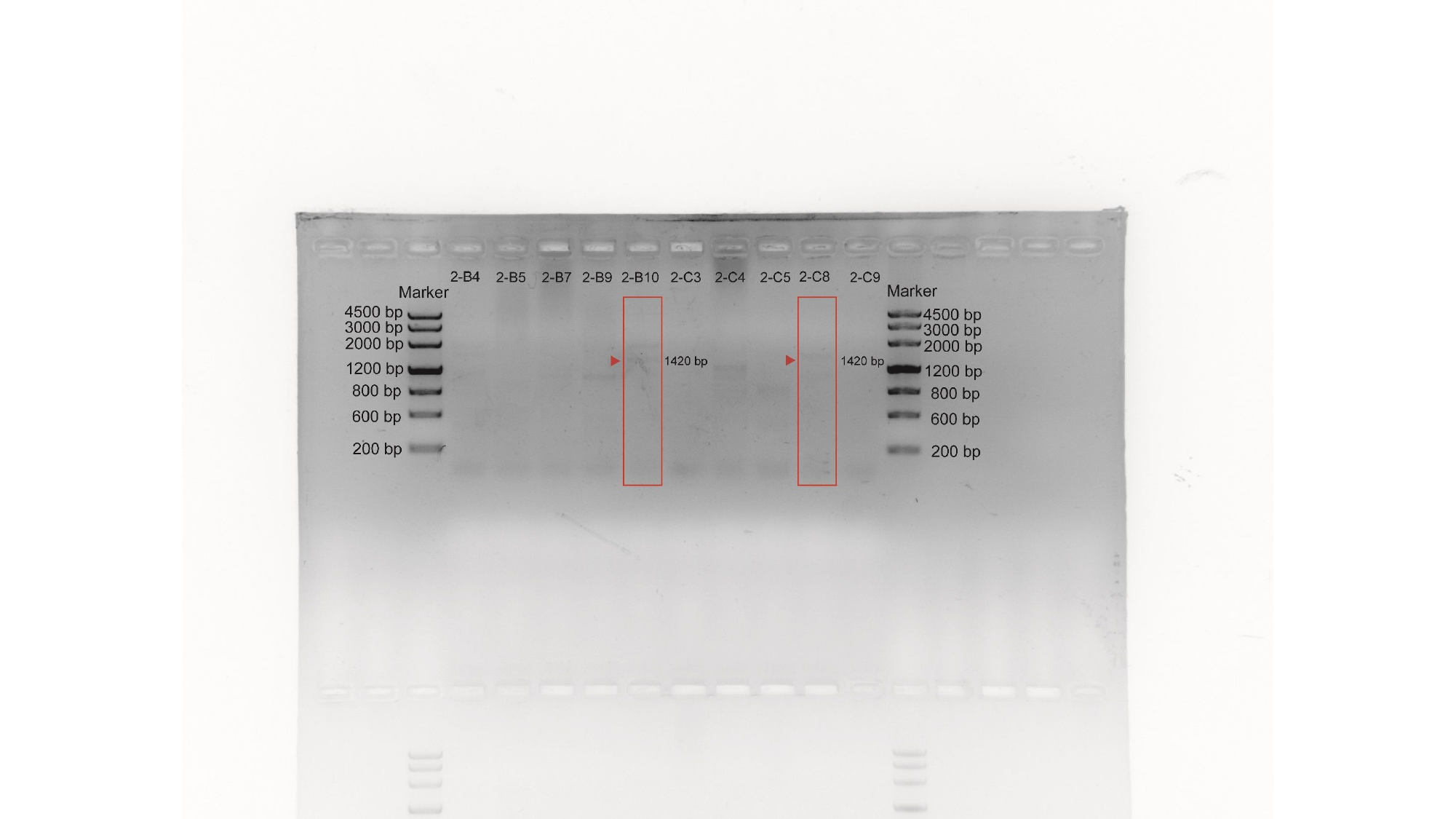

Supplement: Figure 1—figure supplement 1—source data 3. [file elife-78044-fig1-figsupp1-data3.zip › Figure 1-figure supplement 1-source data 3.pptx]

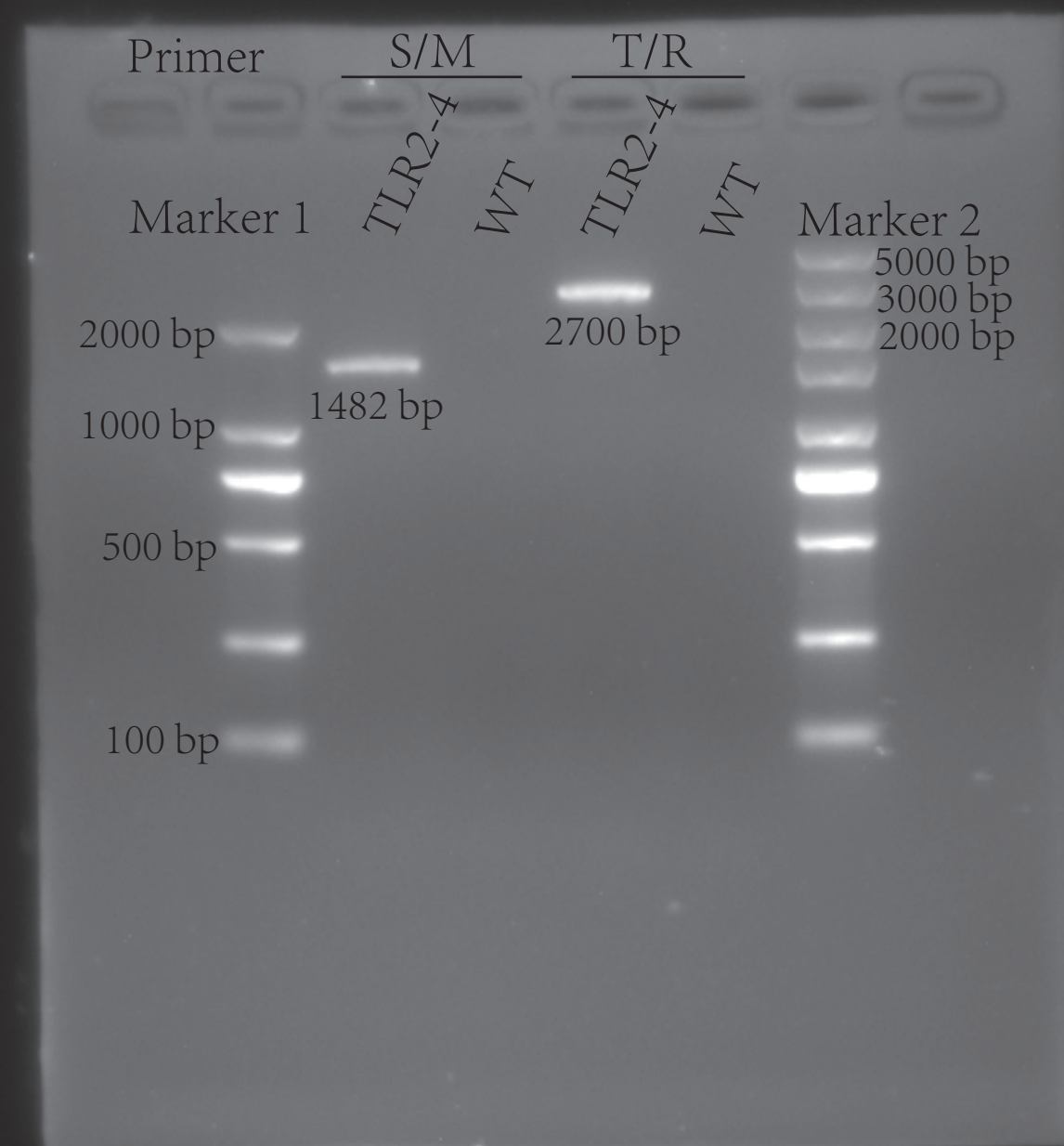

Supplement: Figure 2—source data 1. [file elife-78044-fig2-data1.zip › Figure 2-Source data 1.pdf]

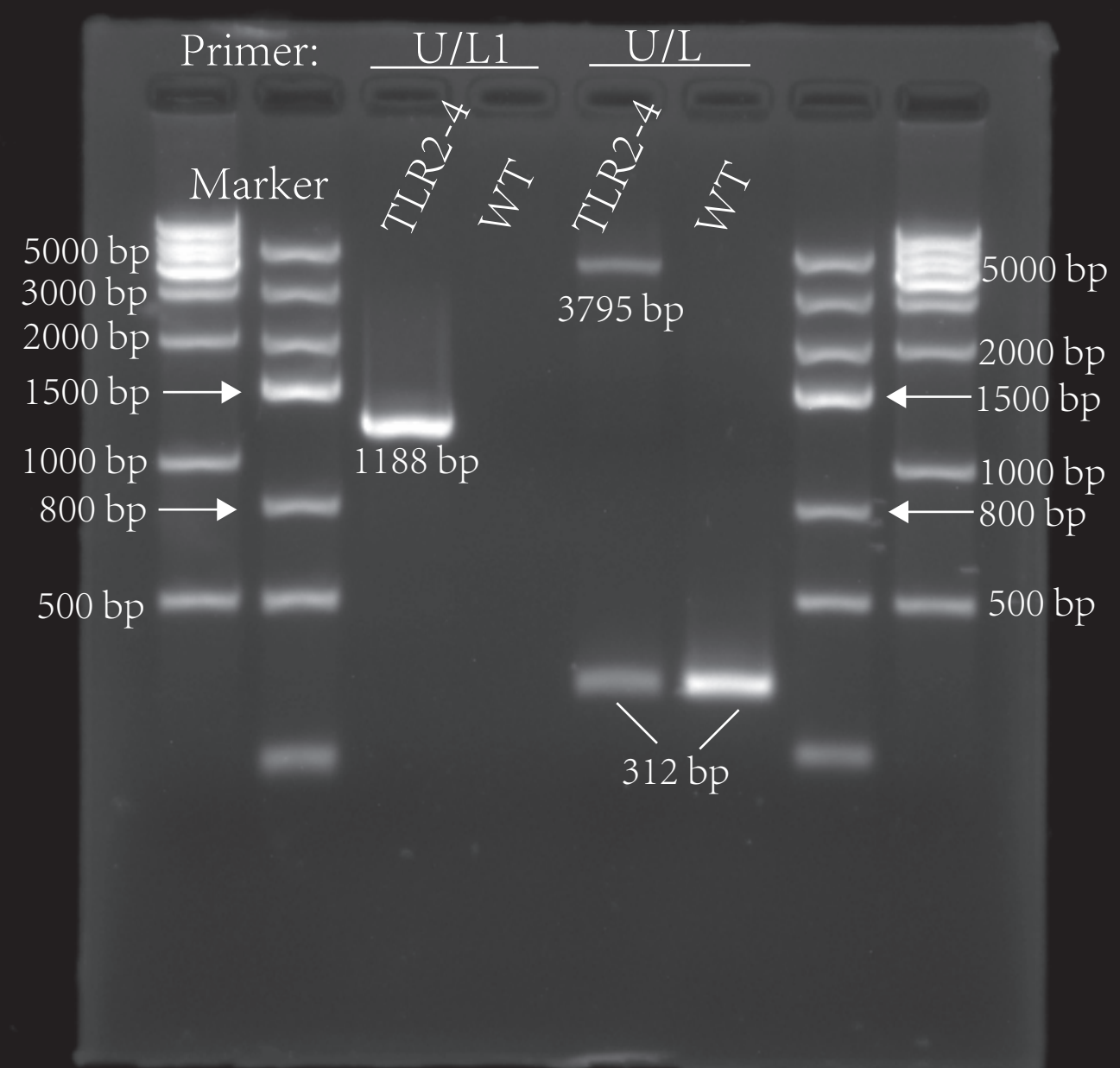

Supplement: Figure 2—source data 2. [file elife-78044-fig2-data2.zip › Figure 2-Source data 2.pdf]

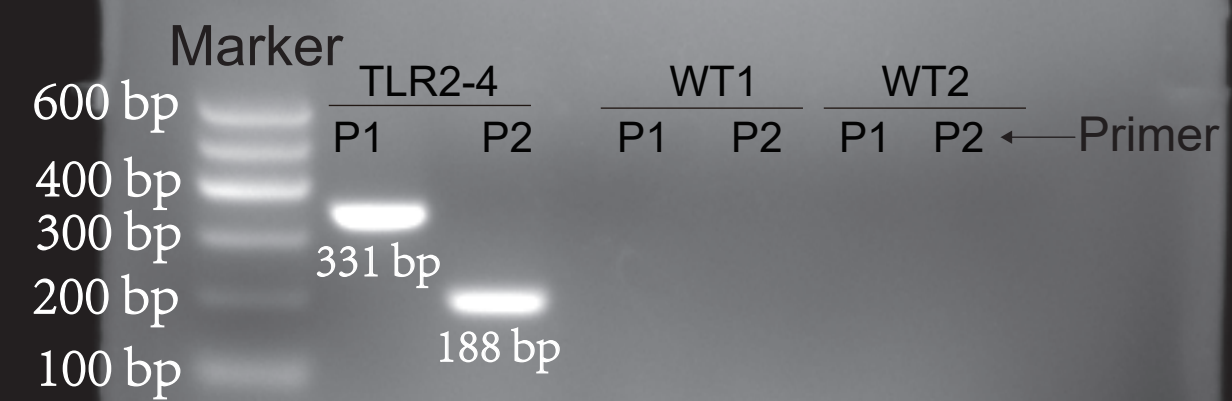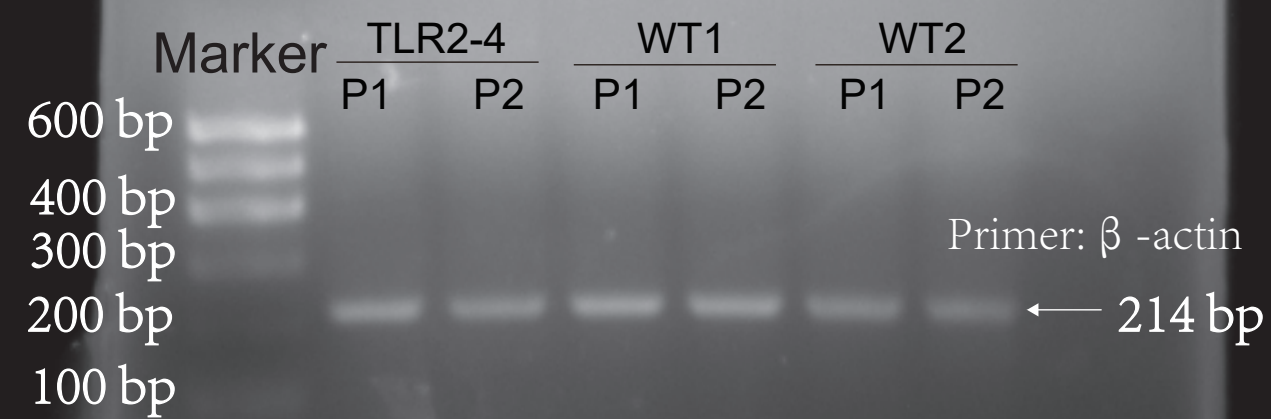

Supplement: Figure 2—source data 3. [file elife-78044-fig2-data3.zip › Figure 2-Source data 3.pdf]

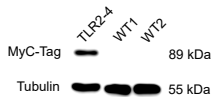

Supplement: Figure 2—source data 4. [file elife-78044-fig2-data4.zip › Figure 2-Source data 4.pdf]

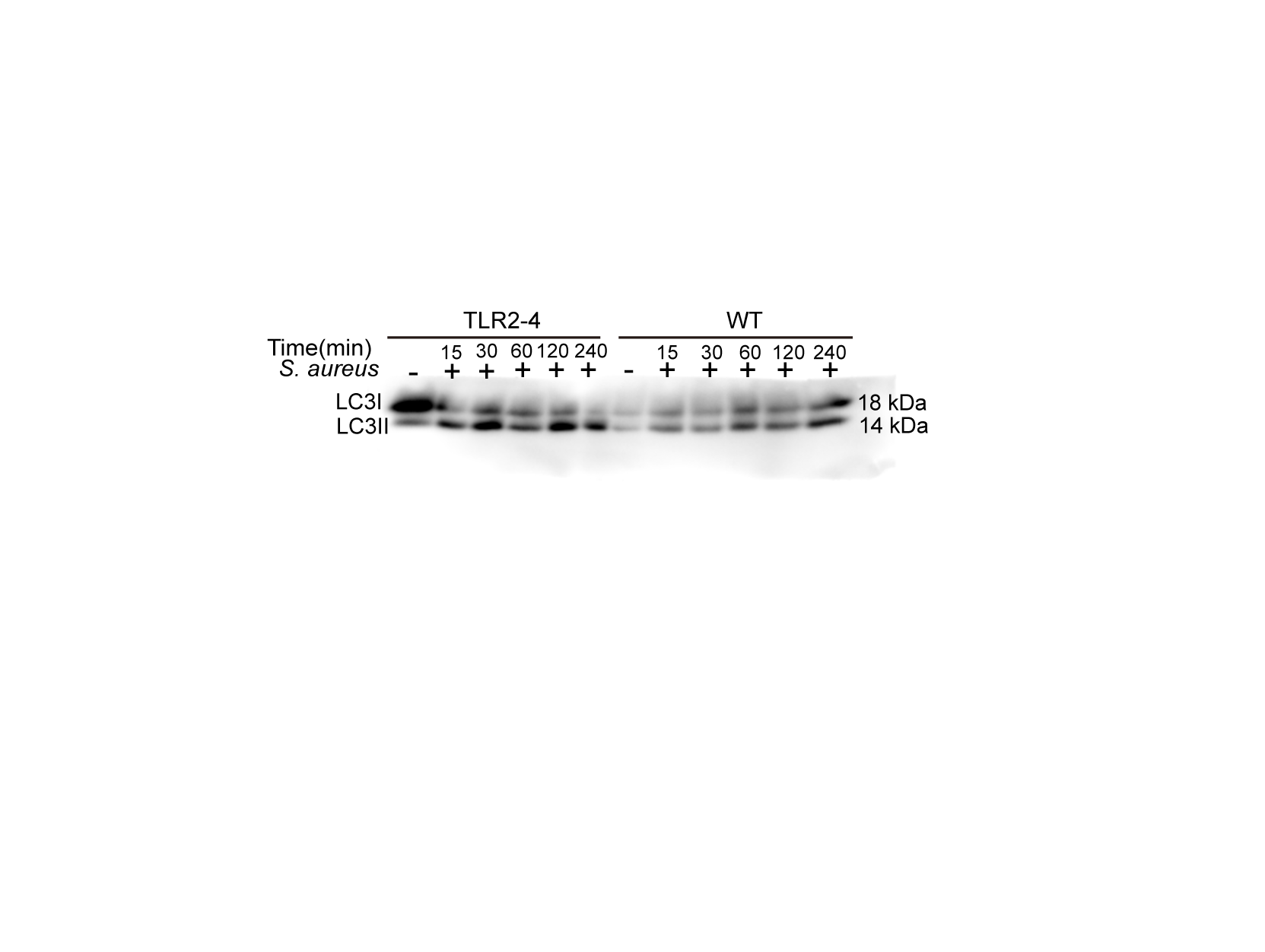

Supplement: Figure 2—figure supplement 1—source data 1. [file elife-78044-fig2-figsupp1-data1.zip › Figure 2-figure supplement 1-source data/Figure 2-figure supplement 1-source data 1(LC3).tif]

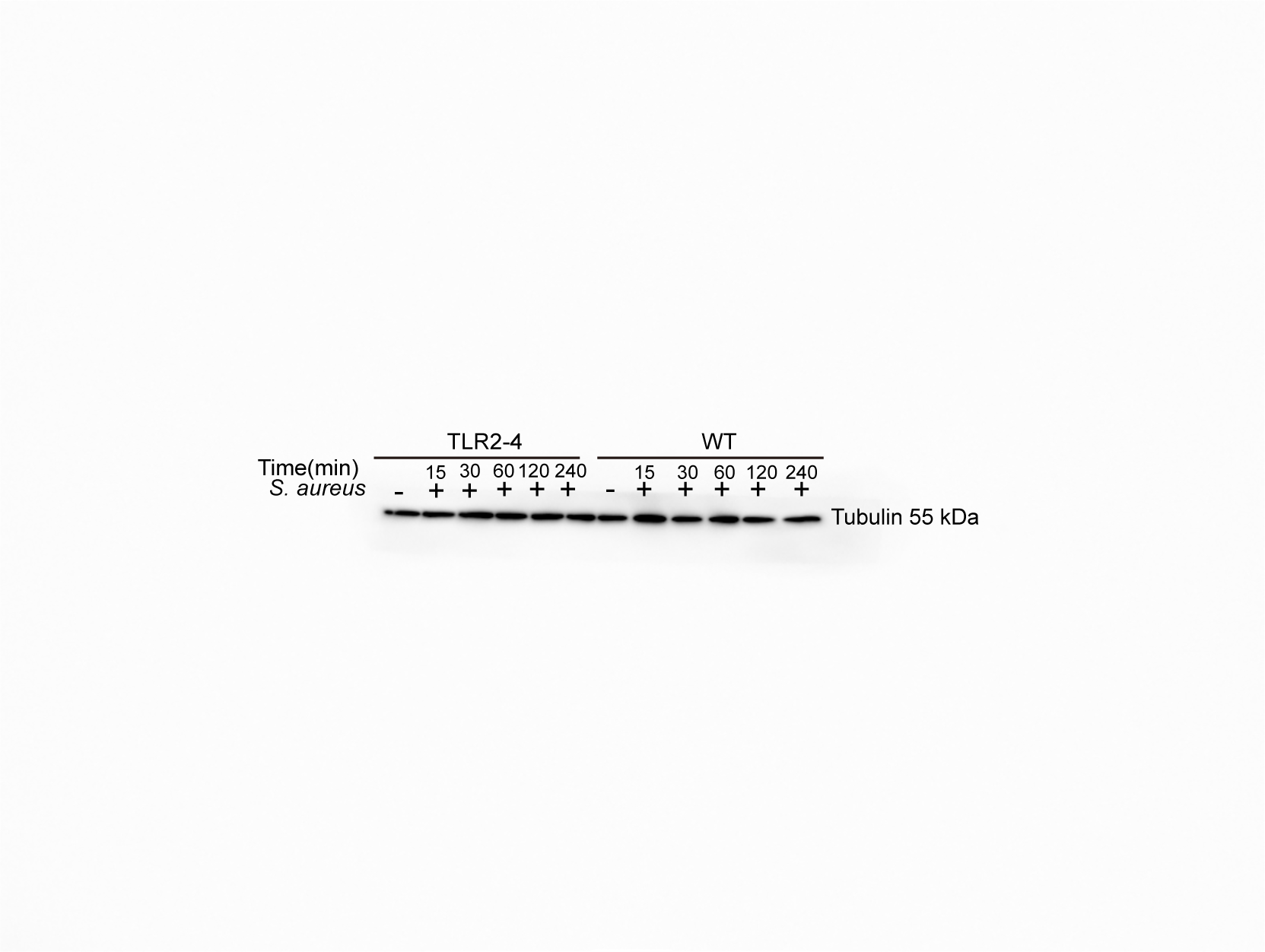

Supplement: Figure 2—figure supplement 1—source data 1. [file elife-78044-fig2-figsupp1-data1.zip › Figure 2-figure supplement 1-source data/Figure 2-figure supplement 1-source data 1(Tubulin).tif]

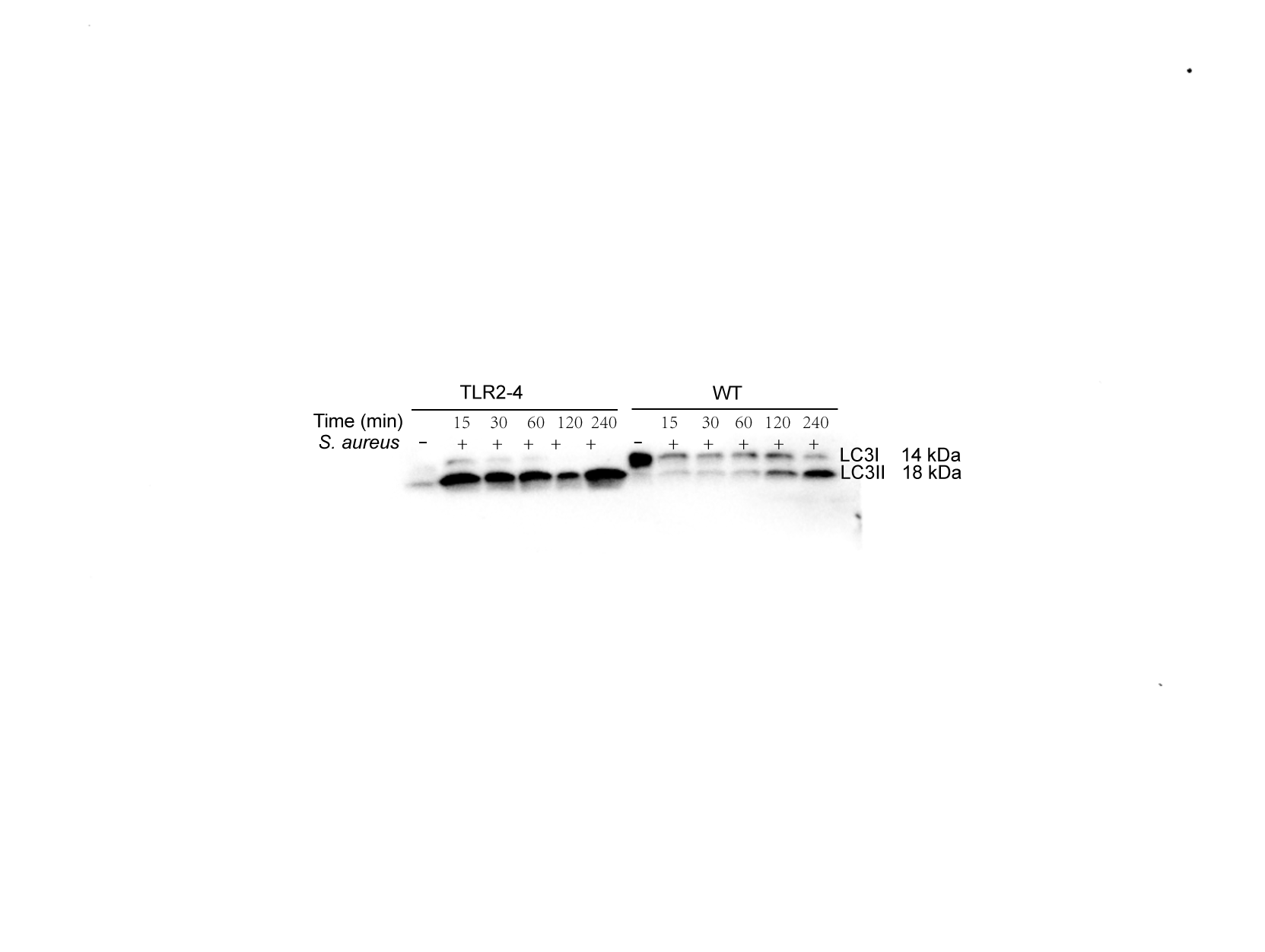

Supplement: Figure 3—source data 1. [file elife-78044-fig3-data1.zip › Figure 3-Source data 1/Figure 1-Source data 1 (LC3).tif]

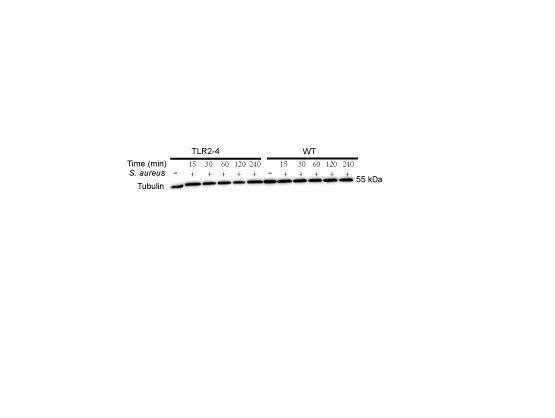

Supplement: Figure 3—source data 1. [file elife-78044-fig3-data1.zip › Figure 3-Source data 1/Figure 1-Source data 1 (Tubulin).tif]

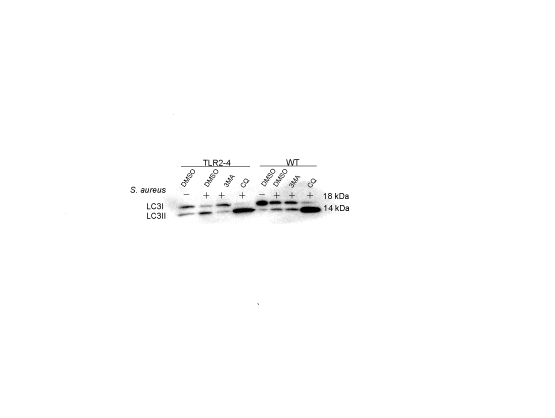

Supplement: Figure 3—source data 2. [file elife-78044-fig3-data2.zip › Figure 3-Source data 2/Figure 1-Source data 2 (LC3).tif]

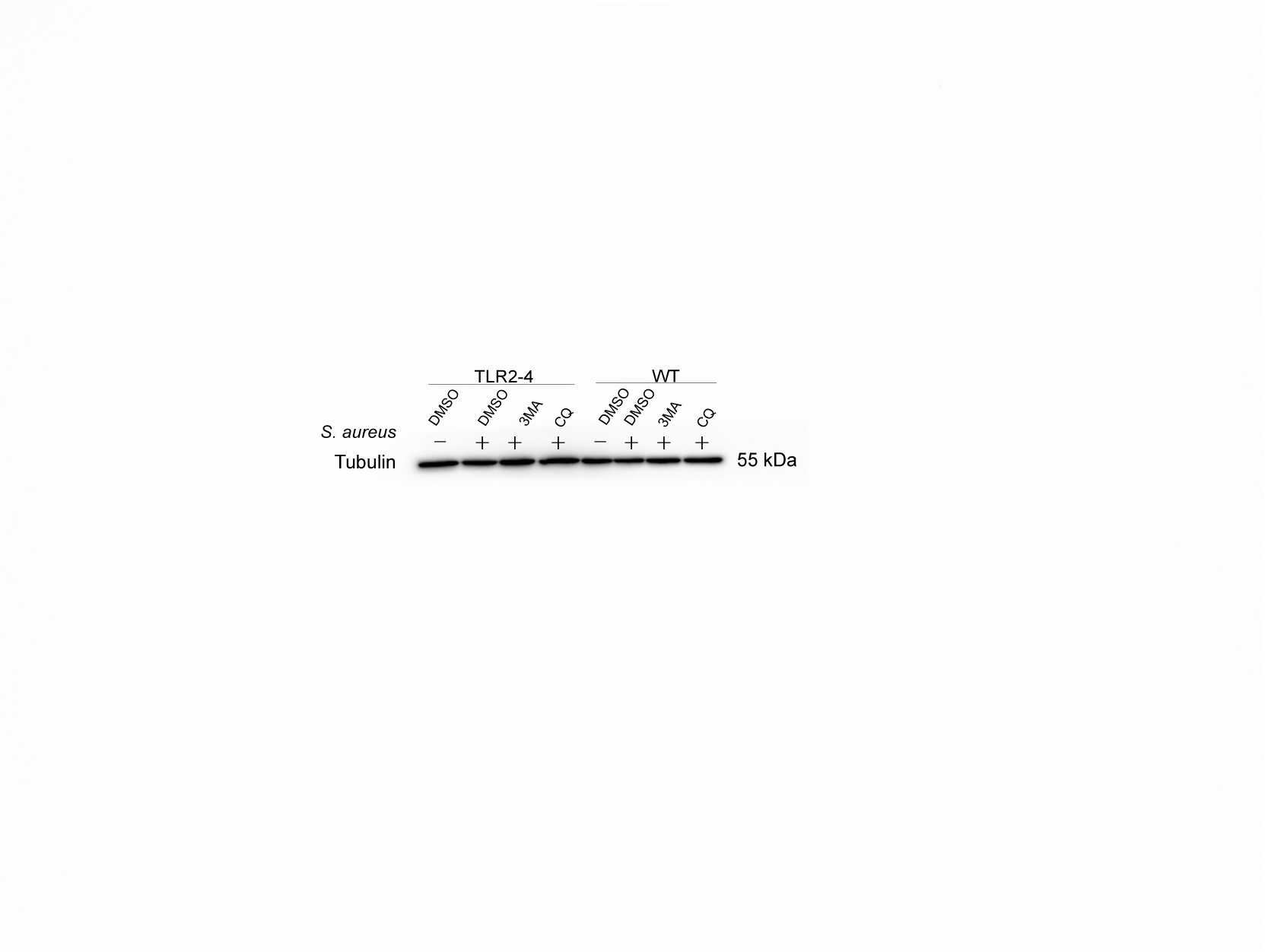

Supplement: Figure 3—source data 2. [file elife-78044-fig3-data2.zip › Figure 3-Source data 2/Figure 1-Source data 2 (Tubulin).tif]

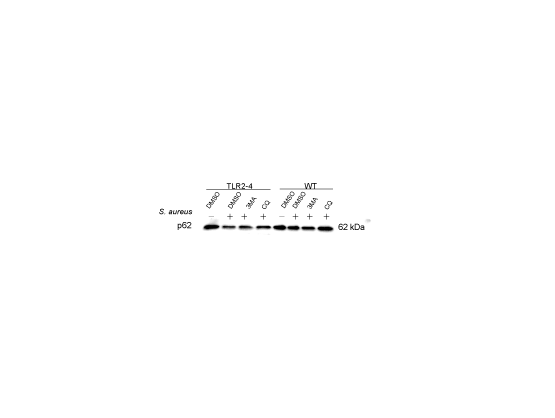

Supplement: Figure 3—source data 2. [file elife-78044-fig3-data2.zip › Figure 3-Source data 2/Figure 1-Source data 2 (p62).tif]

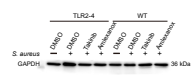

Supplement: Figure 5—source data 1. [file elife-78044-fig5-data1.zip › Figure 5-Source data 1 (GAPDH1).pdf]

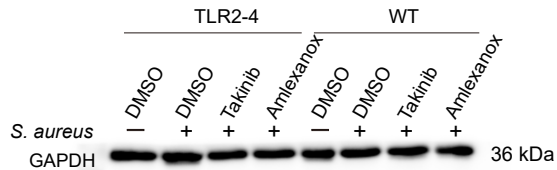

Supplement: Figure 5—source data 1. [file elife-78044-fig5-data1.zip › Figure 5-Source data 1 (GAPDH2).pdf]

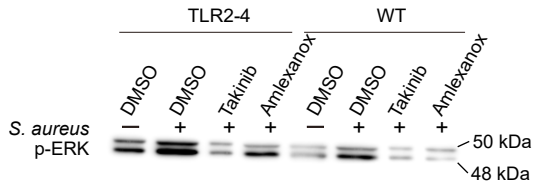

Supplement: Figure 5—source data 1. [file elife-78044-fig5-data1.zip › Figure 5-Source data 1 (p-ERK).pdf]

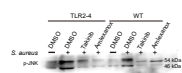

Supplement: Figure 5—source data 1. [file elife-78044-fig5-data1.zip › Figure 5-Source data 1 (p-JNK).pdf]

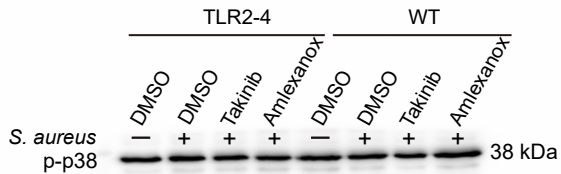

Supplement: Figure 5—source data 1. [file elife-78044-fig5-data1.zip › Figure 5-Source data 1 (p-p38).pdf]

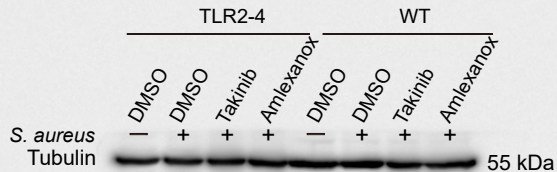

Supplement: Figure 5—source data 1. [file elife-78044-fig5-data1.zip › Figure 5-Source data 1 (Tubulin).pdf]

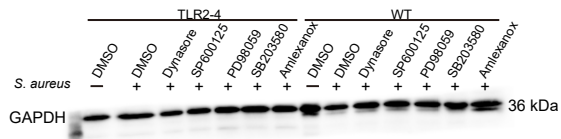

Supplement: Figure 5—source data 2. [file elife-78044-fig5-data2.zip › Figure 5-Source data2 (GAPDH).pdf]

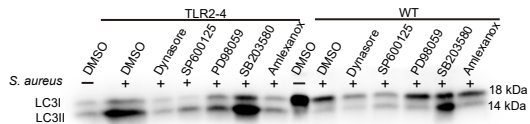

Supplement: Figure 5—source data 2. [file elife-78044-fig5-data2.zip › Figure 5-Source data2 (LC3).pdf]

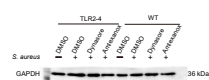

Supplement: Figure 5—source data 3. [file elife-78044-fig5-data3.zip › Figure 5-Source data3 (GAPDH).pdf]

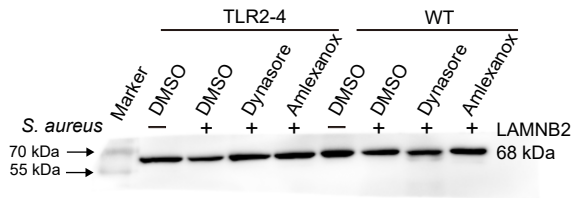

Supplement: Figure 5—source data 3. [file elife-78044-fig5-data3.zip › Figure 5-Source data3 (LAMNB2).pdf]

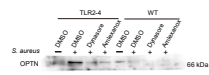

Supplement: Figure 5—source data 3. [file elife-78044-fig5-data3.zip › Figure 5-Source data3 (OPTN).pdf]

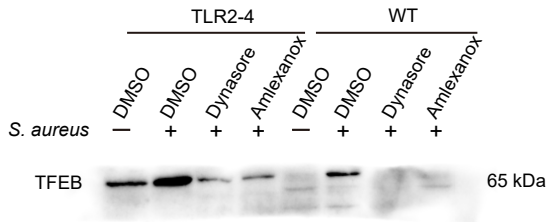

Supplement: Figure 5—source data 3. [file elife-78044-fig5-data3.zip › Figure 5-Source data3 (TFEB).pdf]

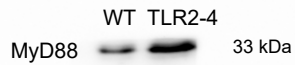

Supplement: Figure 5—figure supplement 1—source data 1. [file elife-78044-fig5-figsupp1-data1.zip › Figure 5-figure supplement 1-source data 1(MyD88).pdf]

WT TLR2-4  
Tubulin 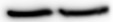 55 kDa

Supplement: Figure 5—figure supplement 1—source data 1. [file elife-78044-fig5-figsupp1-data1.zip › Figure 5-figure supplement 1-source data 1(Tubulin).pdf]

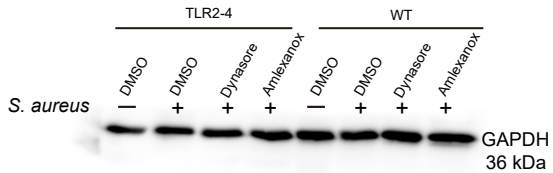

Supplement: Figure 5—figure supplement 1—source data 2. [file elife-78044-fig5-figsupp1-data2.zip › Figure 5-figure supplement 1-source data 2(GAPDH).pdf]

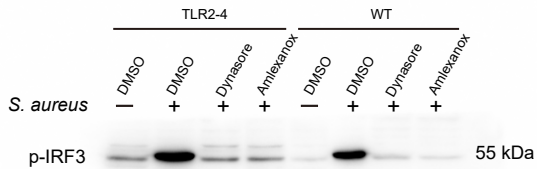

Supplement: Figure 5—figure supplement 1—source data 2. [file elife-78044-fig5-figsupp1-data2.zip › Figure 5-figure supplement 1-source data 2(p-IRF3).pdf]

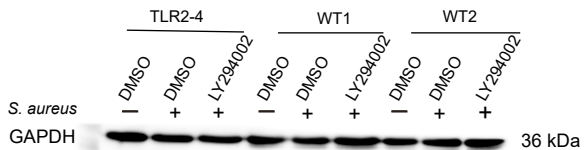

Supplement: Figure 5—figure supplement 1—source data 3. [file elife-78044-fig5-figsupp1-data3.zip › Figure 5-figure supplement 1-source data 3 (GAPDH2).pdf]

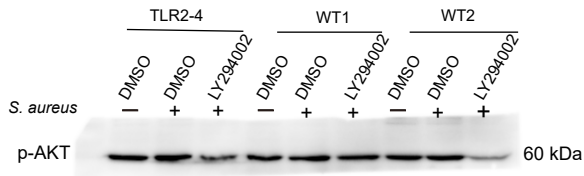

Supplement: Figure 5—figure supplement 1—source data 3. [file elife-78044-fig5-figsupp1-data3.zip › Figure 5-figure supplement 1-source data 3 (p-AKT).pdf]

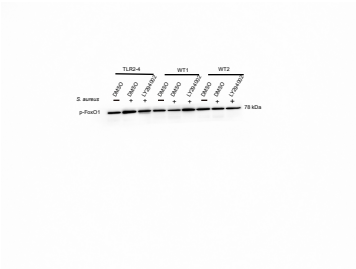

Supplement: Figure 5—figure supplement 1—source data 3. [file elife-78044-fig5-figsupp1-data3.zip › Figure 5-figure supplement 1-source data 3 (p-FoxO1).pdf]

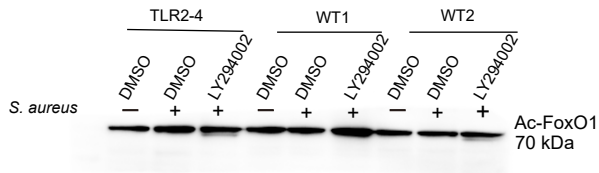

Supplement: Figure 5—figure supplement 1—source data 3. [file elife-78044-fig5-figsupp1-data3.zip › Figure 5-figure supplement 1-source data 3(Ac-FoxO1).pdf]

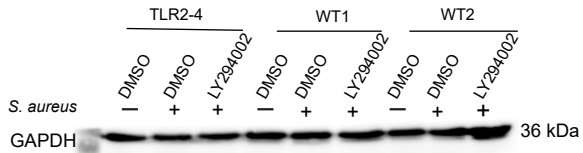

Supplement: Figure 5—figure supplement 1—source data 3. [file elife-78044-fig5-figsupp1-data3.zip › Figure 5-figure supplement 1-source data 3(GAPDH1).pdf]

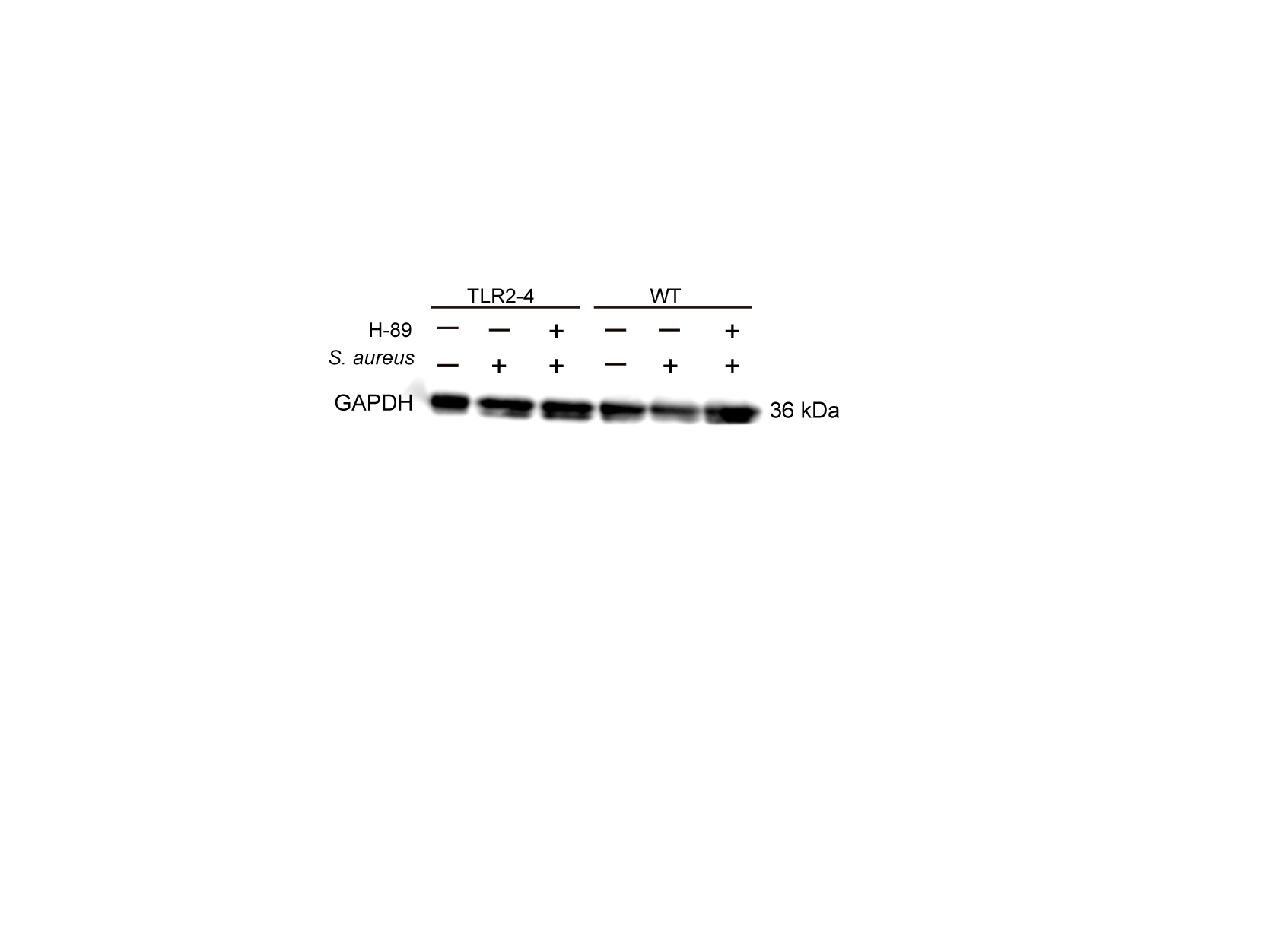

Supplement: Figure 6—source data 1. [file elife-78044-fig6-data1.zip › Figure 6-Source data 1(GAPDH).tif]

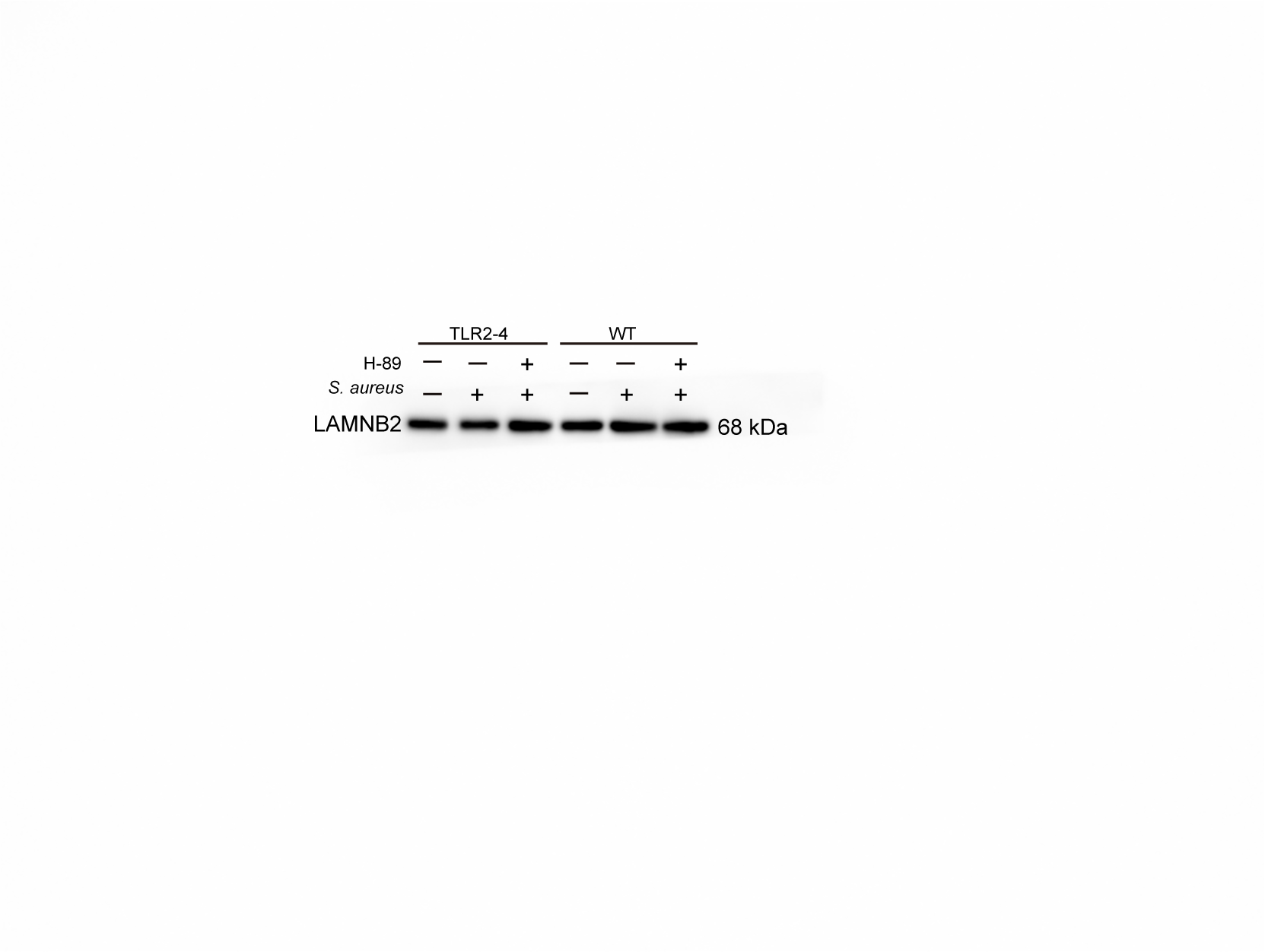

Supplement: Figure 6—source data 1. [file elife-78044-fig6-data1.zip › Figure 6-Source data 1(LAMNB2).tif]

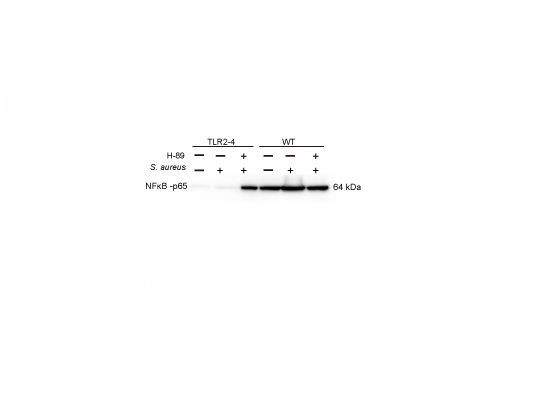

Supplement: Figure 6—source data 1. [file elife-78044-fig6-data1.zip › Figure 6-Source data 1(NF-p65).tif]

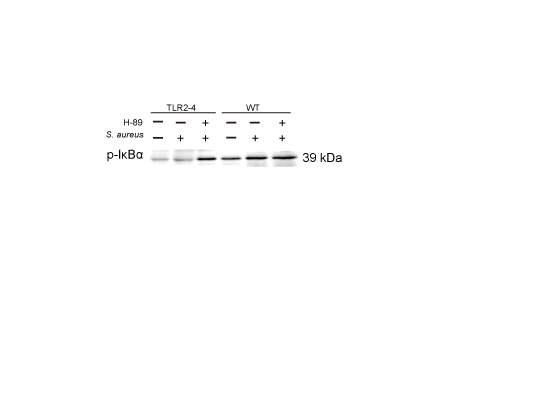

Supplement: Figure 6—source data 1. [file elife-78044-fig6-data1.zip › Figure 6-Source data 1(p-I).tif]

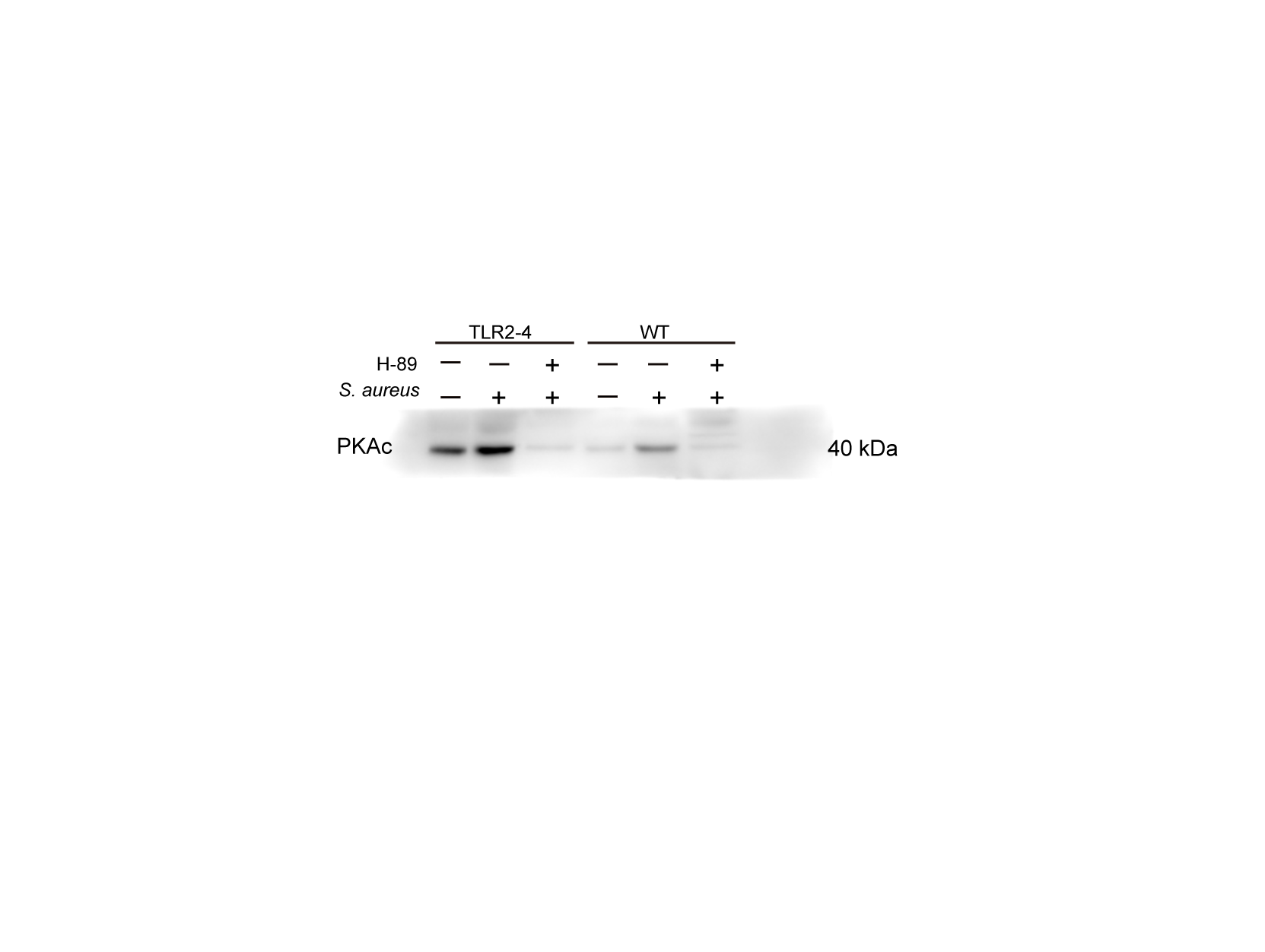

Supplement: Figure 6—source data 1. [file elife-78044-fig6-data1.zip › Figure 6-Source data 1(PKAc).tif]

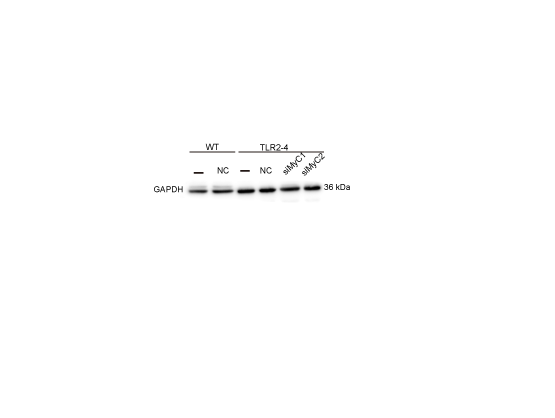

Supplement: Figure 6—source data 2. [file elife-78044-fig6-data2.zip › Figure 6-Source data 2(GAPDH).tif]

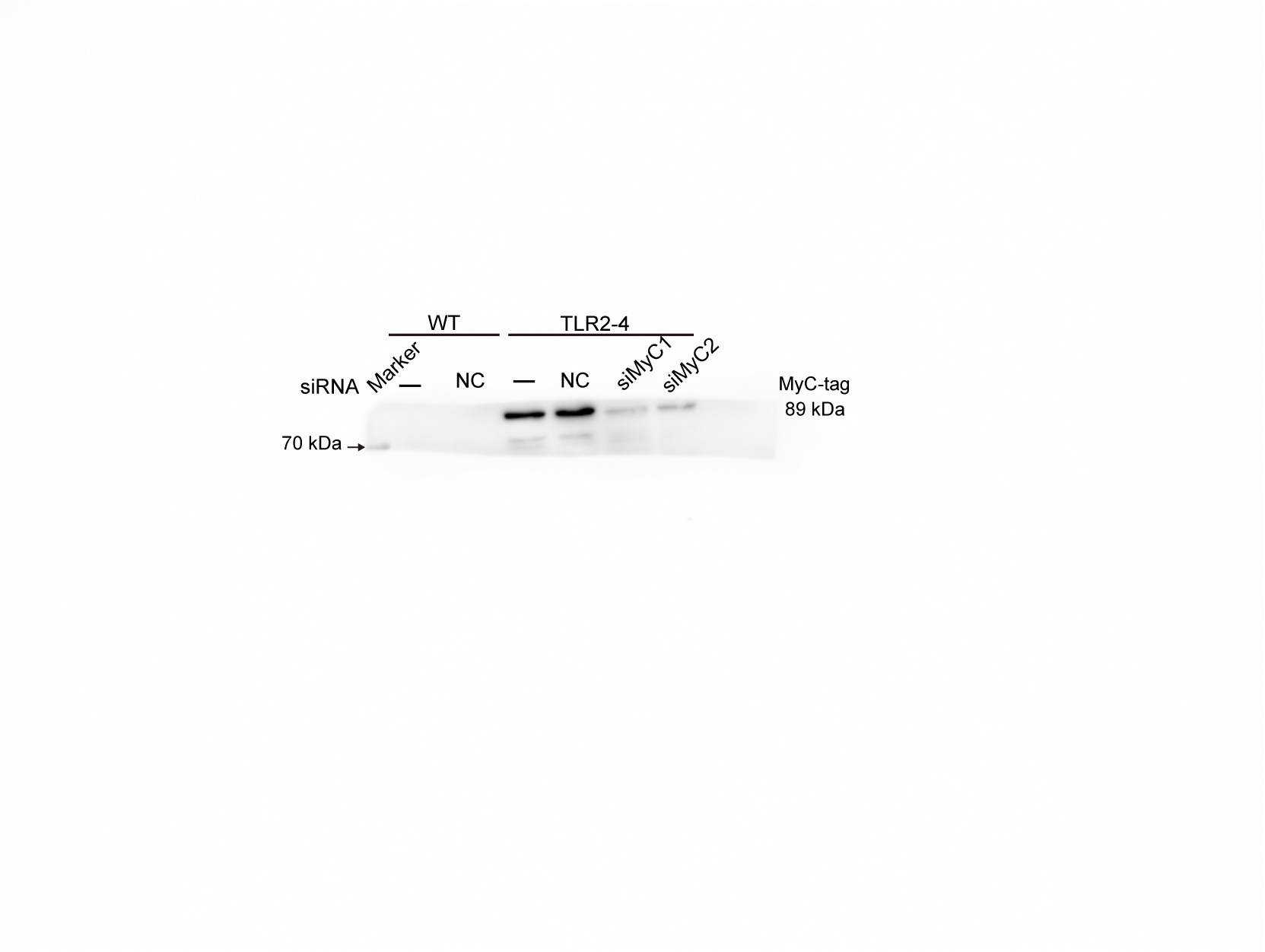

Supplement: Figure 6—source data 2. [file elife-78044-fig6-data2.zip › Figure 6-Source data 2(MyC-Tag).tif]

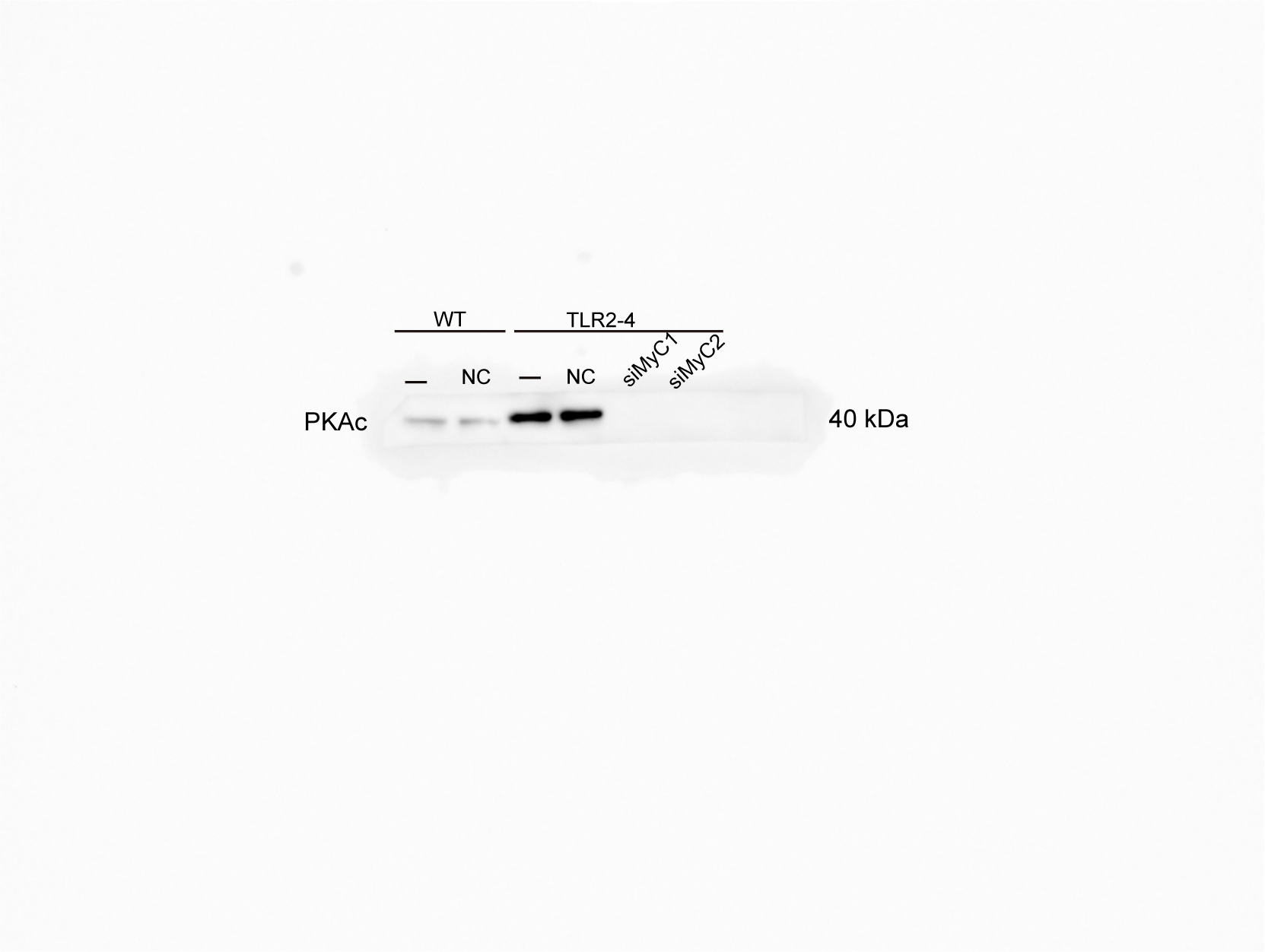

Supplement: Figure 6—source data 2. [file elife-78044-fig6-data2.zip › Figure 6-Source data 2(PKAc).tif]

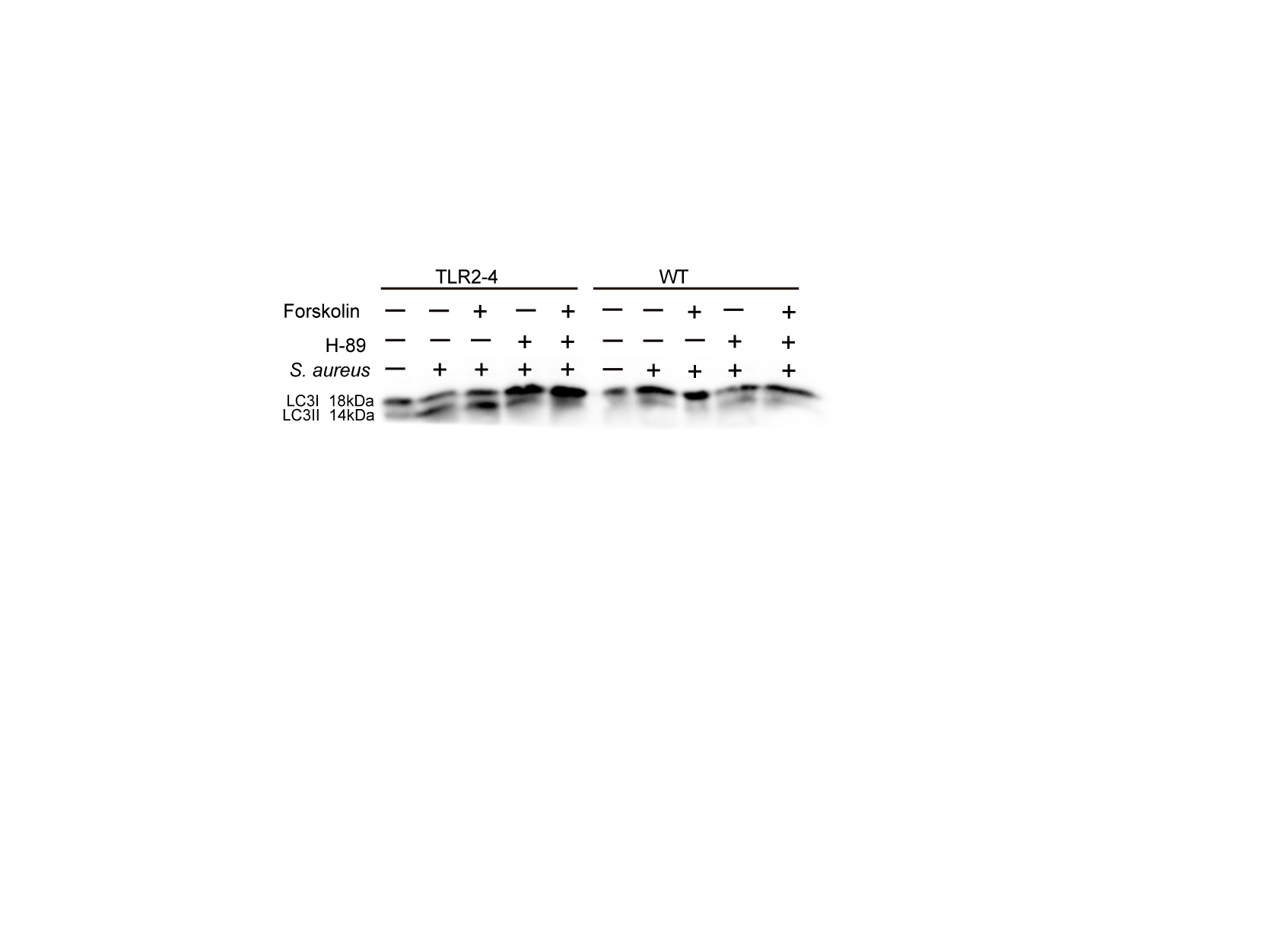

Supplement: Figure 6—source data 3. [file elife-78044-fig6-data3.zip › Figure 6-Source data 3(LC3).tif]

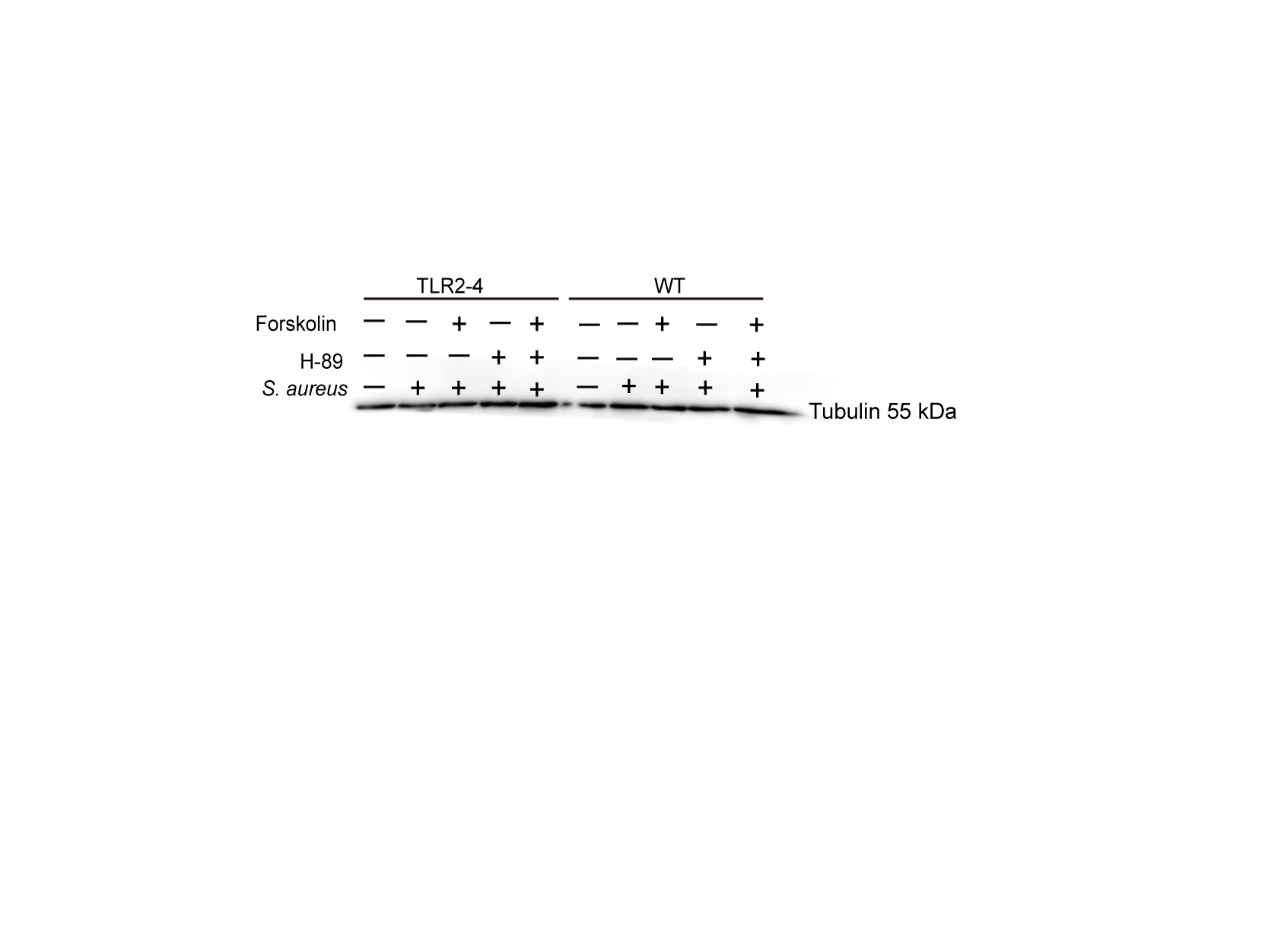

Supplement: Figure 6—source data 3. [file elife-78044-fig6-data3.zip › Figure 6-Source data 3(Tubulin).tif]
