# Supplementary material for: Domain fusion TLR2-4 enhances the autophagy-dependent clearance of Staphylococcus aureus in the genetic engineering goat
Source: eLife. 2022 Jun 28;11:e78044. doi: 10.7554/eLife.78044 (PMC9239677; doi:10.7554/eLife.78044)
Supplement: Supplementary file 1. [file elife-78044-supp1.docx]

**Supplementary file 1. Quantification and statistical analysis.**

The number of independent biological repeats (*n*) is shown in the figure legends.

*P* values are shown below.

|  |  | WT+siTLR2+P2C | WT+siTLR2+P3C | WT+siTLR2+*S. aureus* |
| --- | --- | --- | --- | --- |
|  |  | vs | vs | vs |
|  |  | TLR2-4+siTLR2+P2C | TLR2-4+siTLR2+P3C | TLR2-4+siTLR2+*S. aureus* |
| Fig.1F | IL-8 | <0.0001 | <0.0001 |  |
|  | IL-6 | <0.0001 | <0.0001 |  |
|  | IL-1β | <0.0001 | <0.0001 |  |
| Fig.1G | IL-8 |  |  | <0.0001 |
|  | IL-6 |  |  | 0.0001 |
|  | IL-1β |  |  | 0.10 |

|  |  | TLR2-4 | TLR2-4 | TLR2-4 |
| --- | --- | --- | --- | --- |
|  |  | vs | vs | vs |
|  |  | WT1 | WT2 | WT |
| Fig.3B | 15 min | 0.060 | 0.87 |  |
|  | 30 min | 0.045 | 0.074 |  |
|  | 60 min | <0.0001 | 0.03 |  |
|  | 120 min | 0.0019 | 0.00059 |  |
| Fig.3C | 4 h | <0.0001 | 0.00041 |  |
|  | 8 h | 0.0029 | 0.00062 |  |
|  | 12 h | 0.045 | 0.0041 |  |
|  | 24 h | 0.0018 | 0.00016 |  |
| Fig.3I | 0 h |  |  | <0.0001 |
|  | 1 h |  |  | <0.0001 |
|  | 2 h |  |  | <0.0001 |
|  | 4 h |  |  | <0.0001 |
|  | 8 h |  |  | <0.0001 |

|  |  | TLR2-4 | WT1 | WT2 |
| --- | --- | --- | --- | --- |
|  |  | vs | vs | vs |
|  |  | TLR2-4+3-MA | WT1+3-MA | WT2+3-MA |
| Fig.3H | 8 h | <0.0001 | 0.025 | 0.038 |
|  | 12 h | 0.0049 | 0.16 | 0.48 |
|  | 24 h | 0.0023 | 0.085 | 0.020 |

|  |  | TLR2-4+*S. aureus* | TLR2-4+*S. aureus* |
| --- | --- | --- | --- |
|  |  | vs | vs |
|  |  | WT1+*S. aureus* | WT2+*S. aureus* |
| Fig.6A | ATG 5 | 0.013 | 0.017 |
|  | ATG 12 | 0.022 | <0.0001 |

|  |  | TLR2-4+*S. aureus* | TLR2-4+*S. aureus* |
| --- | --- | --- | --- |
|  |  | vs | vs |
|  |  | TLR2-4+*S. aureus*+SP600125 | TLR2-4+*S. aureus*+PD98059 |
| Fig.6B | ATG 5 | 0.00012 | <0.0001 |
|  | ATG 12 | 0.015 | 0.013 |

|  |  | TLR2-4 | TLR2-4 | WT1 con | TLR2-4 con |
| --- | --- | --- | --- | --- | --- |
|  |  | vs | vs | vs | vs |
|  |  | WT1 | WT2 | WT1+ *S. aureus* | TLR2-4+*S. aureus* |
| Fig.6C | con | 0.0061 | 0.039 | 0.016 | 0.033 |
|  | *S. aureus* | 0.022 | 0.033 |  |  |

|  |  | TLR2-4 | TLR2-4+*S. aureus* | TLR2-4+*S. aureus* |
| --- | --- | --- | --- | --- |
|  |  | vs | vs | vs |
|  |  | TLR2-4+*S. aureus* | TLR2-4+*S. aureus*+Forskolin | TLR2-4+*S. aureus*+H-89 |
| Fig.6F | ATG 5 | 0.013 | 0.0048 | 0.014 |
|  | ATG 12 | 0.0015 | 0.038 | 0.0023 |

|  |  | siRNA NC | siRNA NC | siRNA NC |
| --- | --- | --- | --- | --- |
|  |  | vs | vs | vs |
|  |  | siRNA 90 | siRNA 168 | siRNA 221 |
| Fig.S1F | Endogenous TLR2 | 0.074 | <0.0001 | <0.0001 |

|  |  | WT NC+*S. aureus* | TLR2-4 NC+*S. aureus* |
| --- | --- | --- | --- |
|  |  | vs | vs |
|  |  | WT siTLR2+*S. aureus* | TLR2-4 siTLR2+*S. aureus* |
| Fig.S1G | IL-8 | <0.0001 |  |
|  | IL-6 | 0.0081 |  |
|  | IL-1β | 0.00030 |  |
| Fig.S1H | IL-8 |  | 0.0090 |
|  | IL-6 |  | 0.00098 |
|  | IL-1β |  | 0.035 |

|  |  | WT siTLR2+*S. aureus* |
| --- | --- | --- |
|  |  | vs |
|  |  | TLR2-4 siTLR2+*S. aureus* |
| Fig.S3B | IL-6 | 0.036 |
|  | IL-8 | 0.0014 |
|  | TNF-α | <0.0001 |
|  | IL-10 | 0.29 |

|  |  | TLR2-4 vs WT1 | TLR2-4 vs WT2 | TLR2-4 vs WT3 |
| --- | --- | --- | --- | --- |
|  |  |  |  |  |
| Fig.S3C | 15 min | 0.024 | 0.039 |  |
|  | 30 min | 0.00071 | 0.0015 |  |
|  | 60 min | <0.0001 | 0.00011 |  |
|  | 120 min | <0.0001 | <0.0001 |  |
| Fig.S3D | 15 min | <0.0001 | <0.0001 | 0.00012 |
|  | 30 min | <0.0001 | 0.00022 | 0.00045 |
|  | 60 min | <0.0001 | <0.0001 | <0.0001 |
|  | 120 min | <0.0001 | <0.0001 | <0.0001 |
| Fig.S3F | 15 min | 0.37 | 0.55 |  |
|  | 30 min | 0.046 | 0.45 |  |
|  | 60 min | 0.26 | 0.73 |  |
|  | 120 min | 0.00021 | 0.0005 |  |

|  |  | WT vs TLR2-4 |
| --- | --- | --- |
| Fig.S3G | GFP-LC3 | 0.022 |

|  |  | WT+*S. aureus* | WT+*S. aureus* |
| --- | --- | --- | --- |
|  |  | vs | vs |
|  |  | WT+*S. aureus*+SP600125 | WT+*S. aureus*+PD98059 |
| Fig.S5B | ATG 5 | 0.0085 | 0.027 |
|  | ATG 12 | <0.0001 | 0.00015 |
