# Supplementary material for: Domain fusion TLR2-4 enhances the autophagy-dependent clearance of Staphylococcus aureus in the genetic engineering goat
Source: eLife. 2022 Jun 28;11:e78044. doi: 10.7554/eLife.78044 (PMC9239677; doi:10.7554/eLife.78044)
Supplement: Supplementary file 2. — (A) Table displaying the generation of clone goats by nuclear transfer. (B) Primers of crRNA-oligo. (C) Table displaying the sequences of primers in T7 endonuclease 1 (T7E1) assay. (D) All primers used for PCR. (E) Table displaying the primers for real-time reverse transcription PCR (qRT-PCR). [file elife-78044-supp2.docx]

**Supplementary file 2A.** The generation of clone goats by nuclear transfer.

| Experiments | Clone embryos | Recipients | Pregancies | Newborn lambs |
| --- | --- | --- | --- | --- |
| 1 | 65 | 6 | 1 | 1 |
| 2 | 61 | 6 | 1 | 0 |

**Supplementary file 2B.** Sequence of primers of crRNA-oligo.

| Primer | Sequence (5’-3’) |
| --- | --- |
| 1-top | CACCGACACCATACTTATCTGATA |
| 1-bottom | AAACTATCAGATAAGTATGGTGTC |
| 2-top | CACCGACTACACATTATAGATGACT |
| 2-bottom | AAACAGTCATCTATAATGTGTAGTC |
| 3-top | CACCGTAAGTATCAGATGTAACTGA |
| 3-bottom | AAACTCAGTTACATCTGATACTTAC |
| 4-top | CACCGATGTAACTGATGGTATATT |
| 4-bottom | AAACAATATACCATCAGTTACATC |
| 5-top | CACCGCTAGAAATGTACATACTTTG |
| 5-bottom | AAACCAAAGTATGTACATTTCTAGC |
| 6-top | CACCGATGCCATATCAGATAAGTA |
| 6-bottom | AAACTACTTATCTGATATGGCATC |
| 7-top | CACCGTAGTATGACTCTGGCGTTTG |
| 7-bottom | AAACCAAACGCCAGAGTCATACTAC |
| 8-top | CACCGATTGTCTCCCCCTAAGACTC |
| 8-bottom | AAACGAGTCTTAGGGGGAGACAATC |

**Supplementary file 2C.** The primers of T7E1 assay.

| Primer | Sequence (5’-3’) | Applicable targets |
| --- | --- | --- |
| 01-out-U | CACTGGTAAGATGGATCCAACAATCACT | target-1、2、6、7 |
| 03-in-L | CAACTGTCCTTTGCAGAACGATTTC |  |
| 01-in-U | CTATGAAAAGGCCTTCTATGCTCTTAAGG |  |
| 01-in-L | GGAGAATCACCAGCCCAATATACCATC |  |
| 02-out-U | GGCAGCAAGGAATGGTGTCAATC | sgRNA-5 |
| 01-in-L | GGAGAATCACCAGCCCAATATACCATC |  |
| 01-out-U | CACTGGTAAGATGGATCCAACAATCACT |  |
| 02-in-L | GCATTGTCTTTCAGTGTTGCAAGGG |  |
| 01-in-U | CTATGAAAAGGCCTTCTATGCTCTTAAGG | sgRNA-3、4、8 |
| 03-out-L | GGGATTTTGCTTGGATAGGACCA |  |
| 03-in-U | TCTTTCTGGTTCCCGCCACA |  |
| 03-in-L | CAACTGTCCTTTGCAGAACGATTTC |  |

**Supplementary file 2D.** The primers for PCR.

| Primer | Sequence (5’-3’) |
| --- | --- |
| TLR2-U | GCCTCTGA TCAGGCTTCTTC |
| TLR2-L | CCGGTGGCATTCAGAAAGGGA |
| TLR4-U | ATCATCAGCGTGTCGGTTGT |
| TLR4-L | TCAGGTGGAGGTGGTCGCTT |
| U-110 | ATGCCACGTGCTTTGTGGACAGCGTGGGTCTGGGCTGTAATCAGCGTGTTCACGGAAGGAGAGCAGAAACTCATCTCTGAAGAGGATCTGGCCTCTGATCAGGCTTCTTC |
| L-41 | ACAACCGACACGCTGATGATCCGGTGGCATTCAGAAAGGGA |
| T2-U20 | A TGCCACGTGCTTTGTGGAC |
| T4-U20 | TCAGGTGGAGGTGGTCGCTT |
| HA-R-U | GGGTTCA TTTACACAAAACCGAACA TAC |
| HA-R-L | TTAA TGCTGTTGGCTGCAGG |
| HA-L-U | ACTACACATTATAGATGACTGGGCCCAAGCTATGGGGTATCTACTCAATTCA |
| HA-L-L | ATCTATAATGTGTAGTATGACTCTGGCGTT |
| Td-U | ATGGTGAGCAAGGGCGAGGA |
| Td-L | TTAAGATACATTGATGAGTTTGGACAAACCACAACTA |
| Ct-U | CTATGAAAAGGCCTTCTA TGCTCTTAAGG |
| R-U | TTGCTTGGTAAGCGCGGATAT |
| R-L | AGAGACACCGAACCACACGA |
| U | ACAGCCAGTATGAGTGACACC |
| L1 | AGACACCAGTTGGGTCACAAG |
| L | TCCCCCTAAGACTCAGGCATC |
| S | GAGTGAGGCAGGATGGGATT |
| M | GGTCAAGGTAAGGGTCCAACA |
| R | CTTCGAGTATGAGATTGCCCA |
| T | CAGGGGTGAGTATACAAGGC |
| P1-F | TGACTTCCTGTCCTTCACACA |
| P1-R | CTTTACCAGTTCATTCCGCA |
| P2-F | ACGGAAGGAGAGCAGAAAC |
| P2-R | CTGTTGCTGACATAGGTGAT |
| β-actin-F | AGCAAGAGAGGCATCCTGAC |
| β-actin-R | CAGGGGTGTTGAAGGTCTCA |

**Supplementary file 2E.** Primers for qRT-PCR.

| Gene | Primer sequence (5’-3’) | Product size (bp) |
| --- | --- | --- |
| IL8 | Forward: AGAGGTGTGCTTAGACCCCA  Reverse: GGAAGCAATGGAAAAAGGAT | 125 |
| IL6 | Forward: ACACCACCCCAAGCAGACTA  Reverse: GCCTGATTGAACCCAGATTG | 211 |
| IL1β | Forward: GGCAACCGTACCTGAACCCA  Reverse: CCACGATGACCGACACCACC | 206 |
| IL10 | Forward: AACCACAAGTCCGACTCAACGAAG  Reverse: CAGGAAGACCAGGCAACAGAGC | 82 |
| TNF-α | Forward: TCTACTCGCAGGTCCTCTTC  Reverse: GAAGACCCCTCCCTGGTAGAT | 201 |
| ATG5 | Forward: TTCCAACTTGCTTCACCCTG  Reverse: ACTTTGTCAGTTACCAACGTC | 109 |
| ATG12 | Forward: TGCTAAAGGCTGTGGGAGAC  Reverse: CAACTGTTCCGAAGCCACAAG | 128 |
| β-actin | Forward: AGCAAGAGAGGCATCCTGAC  Reverse: CAGGGGTGTTGAAGGTCTCA | 214 |
