# Supplementary material for: Domain fusion TLR2-4 enhances the autophagy-dependent clearance of Staphylococcus aureus in the genetic engineering goat
Source: eLife. 2022 Jun 28;11:e78044. doi: 10.7554/eLife.78044 (PMC9239677; doi:10.7554/eLife.78044)
Supplement: Figure 1—source data 2. [file Figure 1-source data 2 (Tubulin).pdf › Figure 1-source data 2 (Tubulin).pdf]

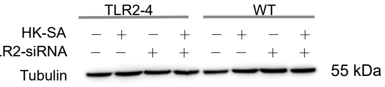
TLR2-4
WT
HK-SA
TLR2-siRNA
Tubulin
55 kDa
